# Effect of Low-Doses of Gamma Radiation on Electric Arc-Induced Long Period Fiber Gratings

**DOI:** 10.3390/s21072318

**Published:** 2021-03-26

**Authors:** Patricia Mesonero-Santos, Ana Fernández-Medina, Luis C. C. Coelho, Duarte Viveiros, Pedro A. Jorge, Tomás Belenguer, Raquel López Heredero

**Affiliations:** 1 Space Optics Department, National Institute for Aerospace Technology (INTA), Carretera de Ajalvir km 4, 28850 Torrejón de Ardoz, Spain; fdezmmab@inta.es (A.F.-M.); belenguer@inta.es (T.B.); lopezhr@inta.es (R.L.H.); 2 Department of Physics and Astronomy of Faculty of Sciences, INESC TEC—Institute for Systems and Computer Engineering, Technology and Science and Faculty for Sciences, University of Porto, 4169-007 Porto, Portugal; lcoelho@inesctec.pt (L.C.C.C.); carlos.d.viveiros@inesctec.pt (D.V.); pedro.jorge@fc.up.pt (P.A.J.)

**Keywords:** gamma irradiation, Electric-Arc Discharge (EAD), Long Period Fiber Grating (LPFG), relaxation

## Abstract

This work presents an experimental study on the effects of gamma radiation on Long Period Fiber Gratings (LPFGs) in a low-dose test campaign to evaluate their eventual degradation. The study was carried out with standard single-mode fibers where the grating was inscribed using the Electric-Arc Discharge (EAD) technique. Before the gamma campaign, a detailed optical characterization was performed with repeatability tests to verify the accuracy of the setup and the associated error sources. The gamma-induced changes up to a dose of 200 krad and the recovery after radiation were monitored with the Dip Wavelength Shift (DWS). The results show that the gamma sensitivity for a total dose of 200 krad is 11 pm/krad and a total DWS of 2.3 nm has been observed with no linear dependence. Post-radiation study shows that recovery from radiation-induced wavelength shift is nearly complete in about 4000 h. Experimental results show that the changes suffered under gamma irradiation of these LPFGs are temporary making them a good choice as sensors in space applications.

## 1. Introduction

Fiber optic sensors are used in a wide range of applications in many fields of industry, engineering, and science offering alternatives to traditional sensors, based on electronic devices, thanks to their characteristics such as electromagnetic immunity, capacity of multiplexing and remote sensing, and power and weight saving; they can be used to measure several physical, chemical, and biological parameters [1,2]. Furthermore, fiber optic sensors have good sensitivity and performance in harsh environments with low and high temperatures, and where ionizing radiation can be present, such as in oil and gas industry, space and nuclear applications [3,4,5,6,7].

This study is focused on sensors based on gratings written in fiber optics. In recent years, this type of sensors have been studied in applications with ionizing radiation, characterizing how radiation affects their properties and its possible application in radiation dosimetry [8]. It is well known from previous studies that the effects of radiation induce changes in fiber gratings. The most investigated effects are the Radiation Induce Attenuation (RIA) and the refractive index change [9,10,11]. It is worth mentioning that, in these studies, direct correlation between these radiation-induced effects was not found. The wavelength shift observed on the reflection or attenuation bands, is related to refractive index change; in the case of Fiber Bragg Gratings (FBGs), it is due to the change in the core effective refractive index and in the case of Long Period Fiber Gratings (LPFGs), it is due to the difference between core and cladding effective refractive indices [12,13,14,15,16].

This type of gratings with longer periods have certain advantages when compared to FBGs; they show greater thermal sensitivity than FBGs and can be also used to measure several parameters at the same time. LPFGs compared to FBGs are sensitive to surrounding refractive index [17,18,19,20] and are easy to manufacture using different techniques as electric arc discharge (EAD) [21,22], CO_2_ laser [23] and femtosecond laser [24] or UV radiation [25]. This work is focused on LPFGs manufactured using the induced EAD technique, which can be applied in a wide range of fibers, including non-photosensitive fibers, standard fibers, and even non-doped fibers with pure silica core, useful, depending on the desired application [22].

Planetary exploration represents one of the greatest challenges of the present and future lander’s science instruments. The main goal of this kind of space missions is to search for extinct or extant life and characterize the environment. Mars and some icy satellites of the giant planets (as Europa, Enceladus, Titan…) are good candidates for having hosted or hosting life. The development of in situ probes used as multi-parameter sensors is of great interest of the space community to study the habitability of the lander impact zone and to identify biomarker [26]. The European Space Agency (ESA) has shown great interest in the development of miniaturized payloads based on optical technologies to detect and quantify biomarkers and to measure temperature with high accuracy for housekeeping purposes and to study the thermo-elastic deformations [27,28]. The payloads must be robust enough to survive the journey and the in situ harsh conditions of the environment, with special care in the design of instrumentation for the icy moons due to long duration of journey and their thin atmosphere. For planetary exploration, sensors based on LPFG can be good candidates thanks to their extremely sensitivity to the external refractive index. In this work, the effects of gamma radiation is studied by submitting the fiber optic samples to doses equivalent to standard space environmental conditions considering the shielding that cover the payloads to prevent from radiation [29,30]. The goal of this work is to evaluate the LPFG degradation as a first step to validate the technology for space applications focusing on low doses

In the present work, an experimental study on gamma radiation sensitivity of LPFGs was manufactured using the EAD technique. Some extra gratings were used as Control samples, preserved from radiation. The gamma radiation campaign was performed in different and discontinuous steps with an average dose rate of 32.95 krad/h and reaching a total dose of 200 krad. A careful analysis was performed to estimate the experimental errors in order to avoid cross-sensitivity effects. The radiation induced effects in terms of Dip Wavelength Shift (DWS) and the relaxation of the samples after irradiation was evaluated to discern if the effects occur permanently or temporarily. Finally, the effects of the gamma radiation in the temperature sensitivity before and after the campaign were also studied.

## 2. Experimental

### 2.1. Samples

In order to evaluate how space radiation environment affects to EAD LPFGs, three units have been manufactured at INESC TEC-Institute for Systems and Computer Engineering, Technology and Science (Porto, Portugal), point-by-point, using a common optical fiber SMF-28e with 4% Ge content in the core, following the procedure in [31]. With a grating period of 410 μm, and an approximate length of 20 mm, the asymmetric 6th order cladding mode LP_1,6_ falls in the desire 1500–1600-nm range window. Two units, LPFG1 and LPFG2, were irradiated and another one (Control LPFG) was preserved for control.

Additionally, two commercial rad-hard FBGs (FBG1 and FBG2 from FemtoFiberTec GmbH) were also irradiated to test their insensitivity acting this way as invariant irradiated samples to discriminate other parameters that could affect the measurements of the LPFGs. Two extra rad-hard FBG units from the same provider were preserved for control (Control FBG1 and Control FBG2, with peak wavelengths very close to FBG1 and FBG2, respectively). The inscription method of these FBGs is based on infrared fs-laser technology, using a point-by-point process, with a grating length of 5.0 mm and a reflectivity of 75.6%. The gratings inscribed are Type II gratings and were written in radiation insensitive fibers, being suitable for harsh environments, such as space applications.

**Figure 1:**
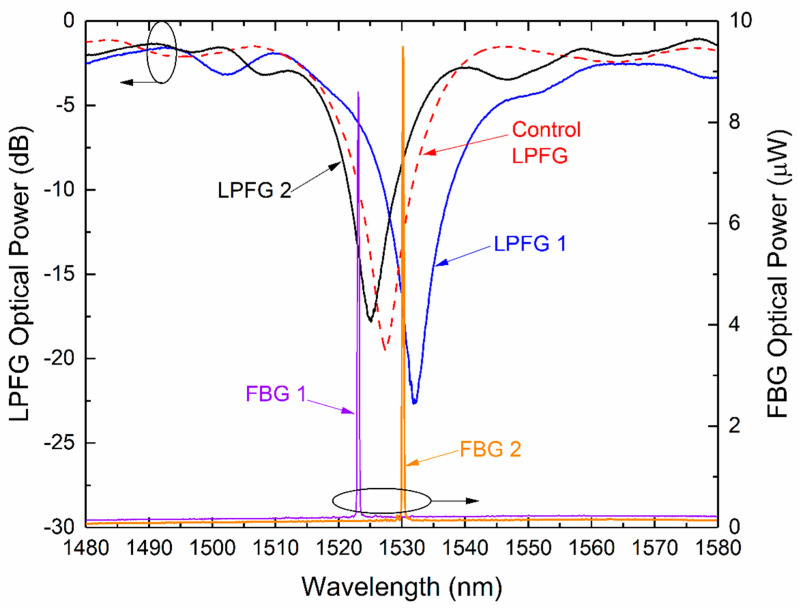
Spectra of the gratings in air at 35 °C: Long Period Fiber Grating (LPFG)1, LPFG2, and Control LPFG measured in transmission mode. Fiber Bragg Grating (FBG)1 and FBG2 measured in reflection mode.

The spectra of all the samples used in this work are represented in Figure 1 (Control FBG1 and Control FBG2 are not depicted to avoid overlapping with FBG1 and FBG2).

### 2.2. Gamma Irradiation Test

**Figure 2:**
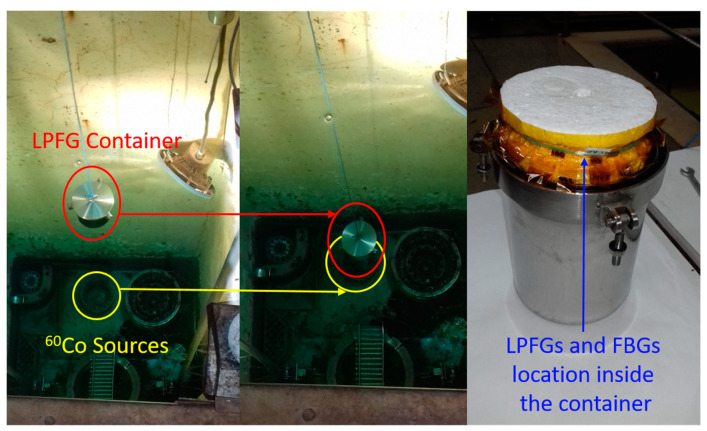
Left: snapshot of mounting process of the container descending inside the pool. Middle: irradiation facility setup during irradiation; view of the container located at the bottom of the pool and the ^60^Co sources in a circular configuration. Right: Waterproof container with LPFGs and FBGs rolled-up inside.

The irradiation of the LPFG sensors has been carried out in the NAYADE facility at Centro de Investigaciones Energéticas Medioambientales y Tecnologicas (CIEMAT) in Spain. A ^60^Co was used as radiation sources placed at the bottom of a pool with a depth of four meters for shielding purposes as shown in Figure 2. The LPFGs and the FBGs sensors were placed in a waterproof container, immersed in water at 18 °C and closely located to the gamma source that is distributed in a circular configuration to assure the homogeneity of the dose received.

At the time of test, the dose rate was evaluated with the Fricke dosimetry technique to calculate the time of irradiation needed for the campaign test. The dose rate was 32.95 krad/h and the dose received by the LPFGs was homogeneous within 94.6%. The samples were irradiated up to 200 krad following four steps at 20 krad, 60 krad, 100 krad, and 200 krad in order to study the response of the LPFGs to different accumulated doses.

### 2.3. Optical Characterization: Repeatability

It is well known that the sensitivity of LPFGs to different external parameters as temperature, strain, and/or external refractive index changes depends on the type and the fabrication process used to inscribe the grating. In this sense, the measurements accuracy can be dependent on the LPFG type.

Repeatability tests (10 measurements) were performed to verify the accuracy of our measurements and associated error sources, as temperature, weight, temporary connectors, and the joint contribution of all these factors in the setup. All these error sources were evaluated by repetitive tests at a temperature of 35 °C ± 0.1 °C with one Control LPFG and with two LPFGs already submitted to the gamma campaign, LPFG1 and LPFG2. LPFG1 was used to measure the Dip Wavelength Shift (DWS) during the test campaign and LPFG2 was used to evaluate additionally if the degradation of the LPFG can affect the error measurements.

**Figure 3:**
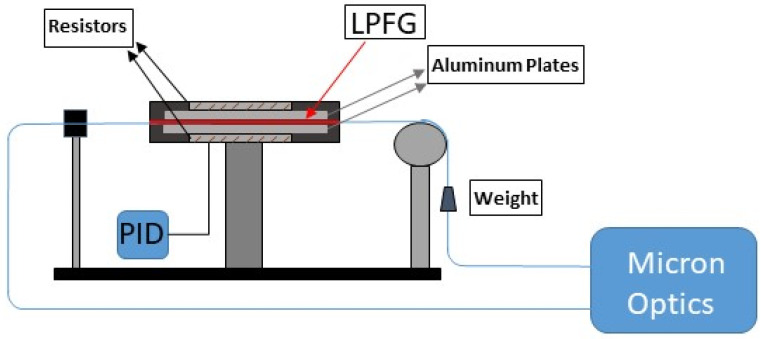
Experimental setup. Proportional Integral Derivative (PID) controller was used for temperature control.

Figure 3 shows the experimental setup used to measure and characterize the optical spectrum and the DWS of the LPFGs. For this purpose, an optical interrogator (Micron Optics, si255) was used with integrated Enlight software for data acquisition. The interrogator light source is a swept wavelength laser with a wavelength range from 1460 nm up to 1620 nm, an acquisition rate of 1 kHz, and a wavelength accuracy of 1 pm, according to the provider.

In order to maintain a stable temperature during the measurements, the LPFGs were placed inside an oven formed by two aluminum plates equipped with resistors. The temperature control was performed by a Proportional Integral Derivative (PID) algorithm that controls the oven temperature by adjusting the parameter control automatically to maintain the set value. For this purpose, a PID controller was used (EZ-ZONE PM, Watlow) and it was programmed to maintain a stable temperature of 35 °C with an oscillation of ±0.1 °C.

The LPFGs were written inducing some strain to the optical fiber by suspending a small weight on one side of the fiber; the same weight needs to be attached after fabrication to recover the dip wavelengths achieved during the manufacturing process. In order to evaluate the experimental error due to the weight, the measurement was performed by mounting and dismounting the weight on the setup.

The samples were rolled-up in the irradiation container without connectors due to the reduced space. Temporary connectors were used during the whole test campaign for optical measurements. The standard deviation of measurements due to mounting and dismounting the connectors from the fiber was analyzed. Amplitude variations were observed as expected, but the study was focused in terms of DWS.

In order to evaluate the contribution to the error due to the temperature oscillations of the PID, the dip wavelengths were monitored during one hour with an acquisition rate of ten measurements per minute. To evaluate the overall contribution of these factors together, ten additional measurements were performed dismounting the whole setup (connectors and weight) at a controlled temperature of 35 °C.

Table 1 shows the standard deviation of all the repetitive measurements considered as the experimental error sources independently and the value obtained for the setup measurements. The results exhibit that the weight mounting/dismounting action is the one that produces more uncertainty in the measurements. The increase of the experimental error of both irradiated LPFGs compared to the values of the Control LPFG, ensure that the weight contribution is the main source of experimental error. In this type of LPFG, the weight can cause deformation of the grating itself and the irradiated samples undergo more this effect due to the handling and rolling several times along the whole gamma campaign. It can be observed that the error due to weight is slightly higher in LPFG2 than in the Control LPFG; LPFG1 shows a greater standard deviation. The values obtained with Control LPFG show that the error due to connectors is of the same order as the one obtained with the weight and the whole setup. Taking into account these values, it can be concluded that great care must be applied when the LPFGs are measured to avoid handling errors.

At the end of the test campaign, a temperature calibrator (Sika TP381165, accuracy 0.01 °C) was used to precisely characterize the response of the FBGs before and after irradiation. The verification of the invariance in their thermal response after irradiation resulted fundamental to discern between the changes observed with the irradiated LPFGs and the experimental errors commented above. The FBGs were placed inside the standard dry block calibrator and were measured in reflection with the optical interrogator during the temperature characterization at an acquisition rate of 100 Hz. The calibrator was programmed with eight temperature steps (−10, 0, 10, 20, 30, 40, 50, and 60 °C). Each step of the calibrator temperature was stable during 20 min within ±0.01 °C before continuing with the next one. The peak wavelength of the FBGs was saved with the interrogator every 10 s during the whole temperature calibration. The mean values and the standard deviation of the measurements were calculated for each step of stabilized temperature. The standard deviation obtained was between 1 and 2 pm.

## 3. Results

The results obtained to evaluate the effect of gamma radiation on LPFG are shown below. Before and after each step of irradiation in the facility, the LPFGs were brought back to the laboratory for measurements and to study the sensitivity of the irradiation-related parameters.

### 3.1. Radiation-Induced Wavelength Shift

**Figure 4:**
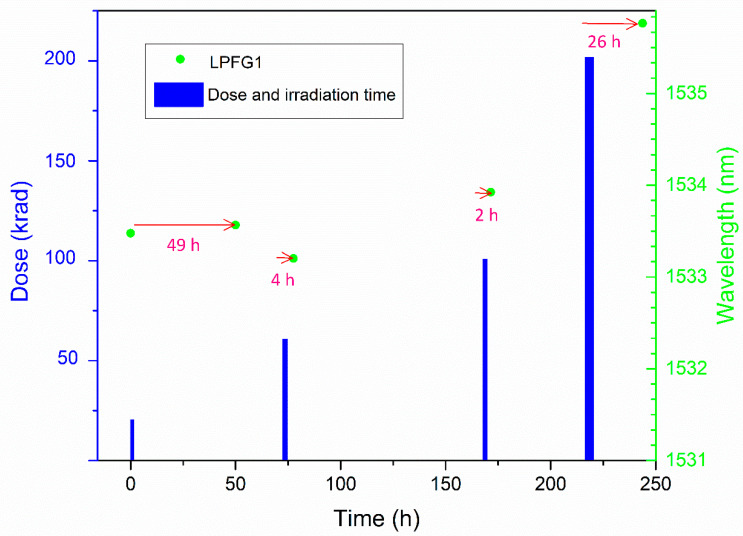
Test campaign irradiation and dip wavelengths measurements versus time. Bars: the width of the bars indicates the exposure time to irradiation for each step (20 krad: 0.62 h; 60 krad: 1.22 h; 100 krad: 1.22 h and 200 krad: 3.03 h). Circles: LPFG1 DWS measurements; the arrows show the time elapsed between irradiation and measurement moment.

The LPFG irradiation was performed in four steps up to a dose of 200 krad, with a dose rate of 32.9 krad/h at room temperature. These sessions were carried out on alternate days due to the availability of the facility and the employees, such as the weekend. Figure 4 shows the steps of irradiation followed during the test campaign; the width of the bars shows the irradiation time starting from zero established from the beginning of the test campaign; the circles show the LPFG1 DWS measurements performed with the optical interrogator and the arrows show the time elapsed between irradiation and measurement moment.

**Figure 5:**
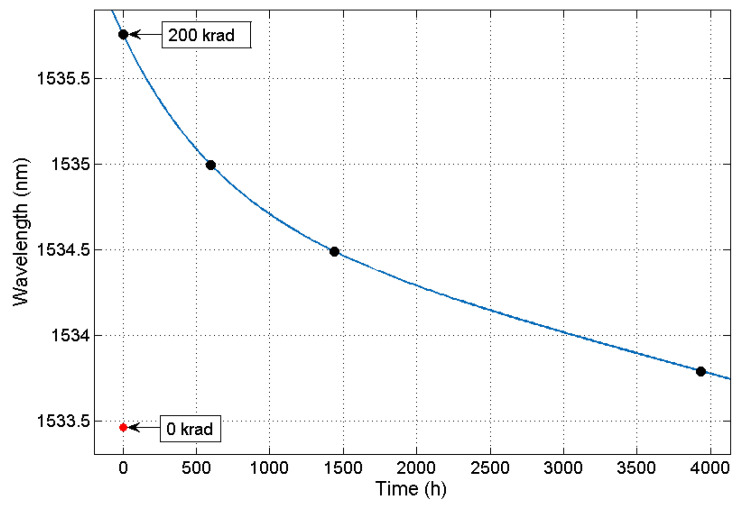
LPFG1 recovery Dip Wavelength after irradiation. Data with 0 krad is depicted for wavelength reference.

After irradiation, LPFG1 was measured to study its relaxation after the last 200 krad step on different days and stored at room temperature (see Figure 5). It can be appreciated that the recovery occurs rapidly in the first hours after irradiation, while the final part of the recovery occurs moderately, presenting an exponential decay. It can be estimated that the LPFG recovery is almost complete around 4000 h after the last step of irradiation, showing a difference of 329 pm with respect to the LPFG measurement before irradiation. The measurements that were carried out in the laboratory both before and after radiation were taken at 35 ± 0.1 °C.

**Figure 6:**
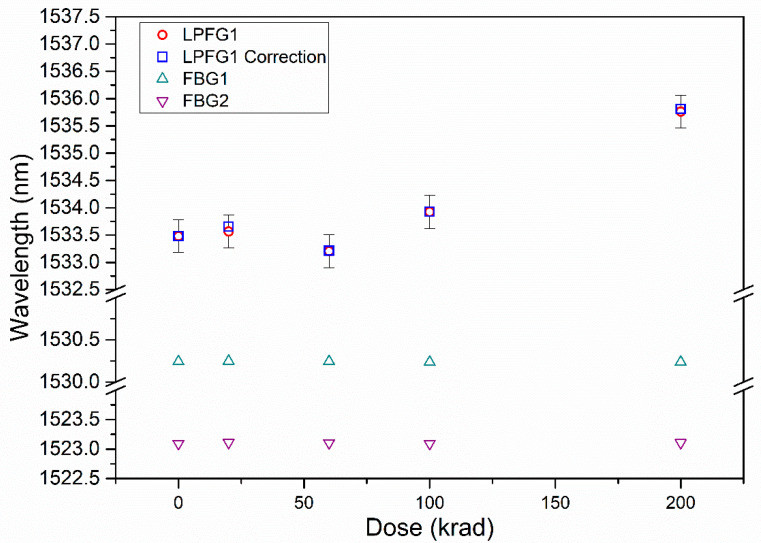
LPFG1 Dip Wavelength with Gamma radiation Dose (circles); LPFG1 data applying measurement time correction due to recovery of LPFG (squares) and data of the rad-hard FBGs (triangles). All the measurements were performed using the optical interrogator.

The study indicates that these LPFGs shows a significant sensitivity to radiation; for a total dose of 200 krad the total wavelength shift obtained is 2.3 nm. It was not observed a great change in transmitted power variation nor attenuation of the dip wavelengths. In order to evaluate the RIA, the use of temporary connectors should be avoided. Figure 6 represents the DWS of LPFG1 during the irradiation campaign and the corrected data due to relaxation considering the time elapsed from irradiation until the measurement (values depicted in arrows in Figure 4). The global radiation sensitivity shown by the LPFG1 up to 200 krad is 11 pm/krad, with no linear dependence. The error bar values are the biggest errors obtained, shown in Table 1 (±0.3 nm). The results shown in Figure 6 are in agreement with other works found in the literature. Table 2 shows the results of LPFGs subjected to gamma irradiation focused on higher absorbed doses than the ones presented in this work. Analyzing the data presented in these studies for doses between 100 and 600 krad (first hours of exposure to radiation), it can be found that the data obtained is in concordance with the results reported in terms of DWS. The LPFG1 shows a higher sensitivity than the EAD LPFGs in Table 2. As it is known, the irradiation parameters and the continuity of the absorbed dose have an influence on the induced changes.

**Table 2:** Comparison data of LPFGs inscribed in SMF-28 subjected to gamma radiation with present work.

Fabrication Method	Dose Rate (krad/h)	Absorbed Dose (krad)	DWS (nm)	Reference
CO_2_	37	108 and 166	1.1 and 1.9	[16]
EAD	18	200 and 600	~1.4 and ~3.0.	[11]
EAD	20	500	~3.0	[12]
EAD	33	200	2.3	Present work

The relaxation study was performed from the total dose accumulated at the end of the irradiation campaign (200 krad). This exponential fit was used to calculate the corrected values in picometers assuming the same behavior for all steps of radiation (20, 60, 100, and 200 krad). A slight difference of a few pm can be observed for all the steps since after a few hours, there are no significant changes. The longest time elapsed between irradiation and measurement was 49 h after the first irradiation step (20 krad) and the applied correction is 89 pm.

The Bragg wavelength of the FBGs measured after each dose step are also shown in Figure 6. The error bars for FBGs data are not depicted since their value is of the order of ±1 pm according to interrogator specifications and the standard deviation obtained with repetitive measurements. It can be considered that the rad-hard FBGs response is not affected by the gamma radiation as expected. It can be noted that the LPFG1 data after 60 krad does not follow the expected behavior; taking into account that the FBG wavelength is within the spectral range expected, it can be assumed that the DWS LPFG1 at this dose presents a deviation due to a handling error when the sample was mounted.

**Figure 7:**
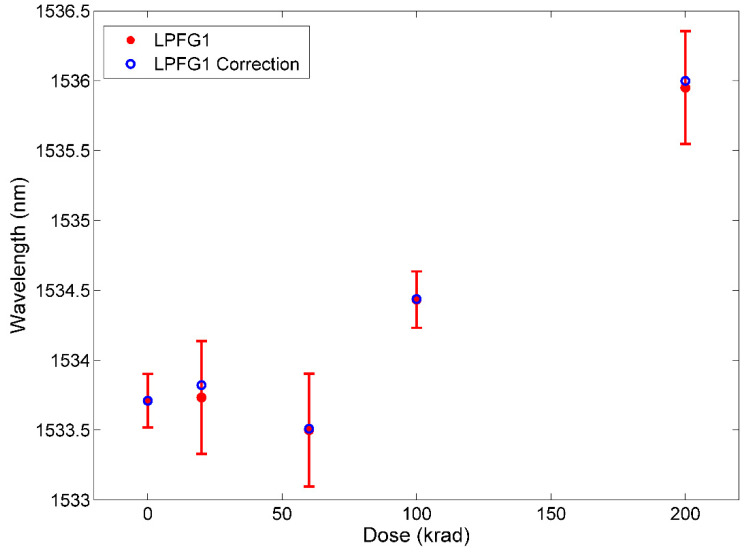
LPFG1 Dip Wavelength with Gamma radiation Dose (in red); LPFG1 data applying measurement time correction due to recovery of LPFG (in blue). These measurements were performed using the Optical Spectrum Analyzer.

The optical characterization after the irradiation was also carried out by measuring the spectra to monitor eventual degradation of the LPFGs optical response covering a wider range, even if the resolution compared to the optical interrogator was smaller. It is important to note that these measurements were carried out just after the ones taken after the optical interrogator. For this purpose, a white light source (Thorlabs SLS201L/M) was used and an Optical Spectrum Analyzer (OSA, model Agilent 86140B) with a resolution of 350 pm for a wavelength range from 1250 nm to 1600 nm. These measurements were taken immediately after measuring with the interrogator, changing the connectors, without altering the rest of the setup. The spectra measurements were made five times. The dip wavelength was located applying a smooth fit and subsequently, a peakfinder function with MatLab^®^. It is important to note that the OSA has lower resolution than of the optical interrogator. Figure 7 shows the DWS measured with OSA and the corrected values from the recovery analysis. As it can be seen, the total DWS is 2.3 nm with both set of values (experimental and corrected), being the same DWS as the value obtained with the optical interrogator (data in Figure 6) and showing the same behavior with respect to the total dose. The error bars represent the value of the standard deviation obtained with five measurements with the OSA (between 200 and 400 pm). It can be observed that the data measurement after 60 krad shows the same discrepancy as in the case of the optical interrogator.

### 3.2. Temperature Sensitivity

It is well known that irradiation process can change the thermo-optic coefficient of the fiber, modifying consequently the temperature sensitivity of the LPFG [14,15]. Temperature sensitivity was analyzed after irradiation for LPFG1. The temperature analysis was also carried out on the Control LPFG with the same characteristics and manufacturing method as LPFG1. In this way, the results can be compared between the temperature sensitivity before and after irradiation for the extended range.

The temperature was studied in a short range due to the fact that in space missions the instruments are usually athermalized and good thermal control is needed for optical payloads [7].

**Figure 8:**
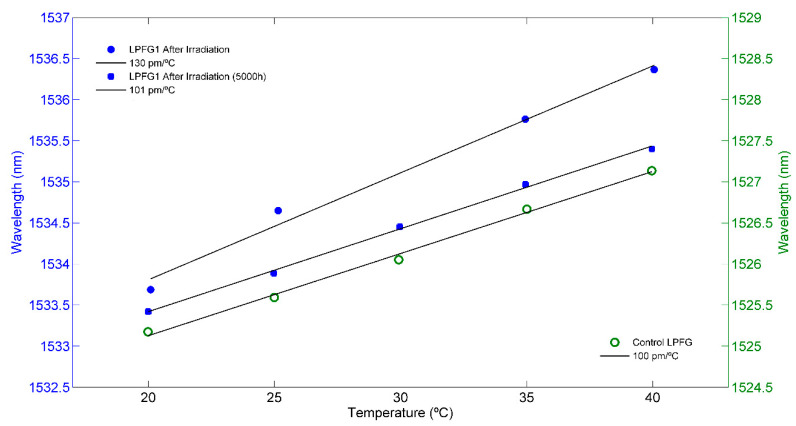
LPFG1 temperature sensitivity data (blue): after irradiation and after recovery (5000 h); Control LPFG temperature sensitivity data (green); linear fits are presented in black.

The Control LPFG shows a temperature sensitivity of 101 pm/°C, which was taken as a reference for the sensitivity to temperature before irradiation. After irradiation, for the same temperature range, LPFG1 shows a temperature sensitivity of 130 pm/°C (see Figure 8). Comparing the results before and after irradiation, LPFG1 shows a great change of thermal sensitivity with a difference of 30 pm/°C after irradiation (30% increase). This increase is higher than the value reported in literature [8] showing a higher change in the thermo-optic coefficient. It can be assumed that the manufacturing process can have a great influence in the thermal response of irradiated samples, as electric current, arc duration, and number of repetitions [21]. The temperature sensitivity of LPFG1 was re-studied around 5000 h after the last irradiation step, assuming that the relaxation is almost complete. The temperature sensitivity of the LPFG1 obtained was 101 pm/°C, corresponding to the sensitivity measured with the Control LPFG.

**Figure 9:**
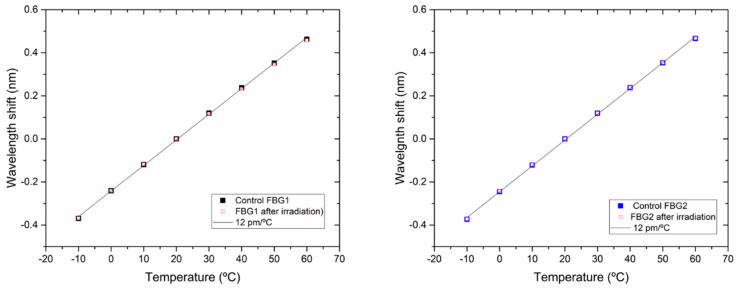
Wavelength shift of the Bragg wavelength when temperature changed from −10 °C to +60 °C: full squares represents the data from Control FBGs; hollow squares represent the FBGs data after irradiation; left: FBG1 and Control FBG1; right: FBG2 and Control FBG2. Linear fits are represented in black lines.

In the case of the rad-hard FBGs, the wavelength shift of the Bragg wavelength due to temperature changes was measured with a temperature calibrator between −10 and +60 °C. Figure 9 shows the data obtained with the Control FBGs preserved from radiation, and the data obtained with the FBGs after the complete gamma test (200 krad). In these figures, the peak wavelength of each FBG at 20 °C was taken as a reference. As it can be appreciated, the FBGs response stayed invariable after irradiation (12 pm/°C) as it was expected due to their characteristics. These values confirm the fact that the variations experimented by the LPFGs in terms of wavelength and in temperature dependency can be attributed to gamma radiation effects.

## 4. Discussion and Conclusions

In the present work, we have studied how the exposure to low doses of gamma radiation affects the behavior of LPFGs written by EAD technique in standard fiber optic, SMF-28, doped with Ge. Most of the studies focused on this area are oriented for high dose applications as nuclear and high energy facilities and on the feasibility to use the LPFGs for radiation dosimetry.

The effects of gamma radiation on the LPFGs have been studied analyzing their DWS and their thermal response. The results obtained show that these LPFGs have a high sensitivity to radiation, since for a total dose of 200 krad, the DWS obtained is 2.3 nm and a total radiation sensitivity of 11 pm/krad, not showing a linear behavior. In addition, it is observed that there is a dependence on the change in the wavelength with the dose, as reported in the literature [16]. These values show a higher radiation sensitivity than the results reported in literature for different types of LPFGs [8] which makes them good candidates as possible dosimetry sensors. The value obtained indicates that the LPFGs fabrication method and the test campaign parameters can influence on their response. In this study the average dose rate was 32.9 krad/h, the samples were exposed to radiation in different and discontinuous steps, and the measurements were not performed in situ.

The measurements were made with an optical interrogator (Micron Optics, si255), with a better resolution compared to a classic Optical Spectrum Analyzer. Nevertheless, the measurements were also performed with an OSA (Agilent 86140B) to compare the results. The results obtained with both instruments are very similar and the DWS for the total radiation dose is the same in both (2.3 nm).

After irradiation, the relaxation of the LPFGs was studied to check if the LPFGs suffer degradation, i.e., whether the radiation-induced changes are permanent or temporary. In view of the results, the EAD LPFGs show a total recovery of the initial parameters after a few months, around 4000 h, stored at room temperature. Therefore, the induced changes are temporary and LPFGs do not undergo degradation.

Rad-hard FBGs were also irradiated to test their insensitivity to radiation, obtaining the expected results and complying with the technical characteristics of the manufacturer. The results show small variations; these values do not follow a typical behavior due to the influence of radiation, but rather present a scattered pattern within the values obtained in the standard deviation of repeatability measurements, being 1 pm. These results verify that the FBGs response does not change after irradiation and result useful to discriminate the radiation effects in the LPFGs from the rest of parameters that can influence the LPFGs measurements. Therefore, the DWS observed in the LPFGs can be attributed to radiation effects.

In relation to the temperature sensitivity, it is known that radiation can induce changes in the thermo-optic coefficient and consequently, changes in the sensitivity to temperature can occur [12]. Sensitivity to temperature was analyzed in the Control LPFG and LPFG1 to study the radiation-induced changes. The Control LPFG showed a sensitivity of 100 pm/°C, while after irradiation, LPFG1 showed a sensitivity to temperature that changed significantly, being 130 pm/°C. It should be noted that the LPFG1 recovered their initial temperature sensitivity (100 pm/°C) once they are totally recovered.

Furthermore, the thermal sensitivity of Control FBGs and the irradiated FBGs was also studied. The verification of the invariance in their thermal response after irradiation confirm that the radiation induced the changes observed with LPFGs.

The LPFGs tested were exposed to low doses of radiation because the study is oriented to their future application in space payloads. The results shown in this study are far from saturation due to the low-doses of gamma radiation applied. The response of the LPFGs used as sensors is always influenced by the temperature and are highly sensitive to strain, bending and curvature. In this sense, calibration in situ is essential to discriminate these effects when measuring other physical or chemical parameters. This fact is not a drawback in space applications where the optical payloads are usually athermalized to guarantee their good performances. Special attention should be taken into account for the packaging design when choosing the materials depending on their Coefficient of Thermal Expansion (CTE).

Our experience with this work shows that extreme care should be taken in consideration when handling the samples. In addition, improvements of packaging to place the LPFGs should be designed to compensate the temperature effects and to avoid the greatest experimental errors observed in this campaign. Moreover, in situ tests during irradiation will give better knowledge of their behavior and allow a deep study of RIA and DWS for low-doses. Our future works will focus on the study of the relaxation for different total accumulated doses for full understanding the process involved in the recovery; this a key aspect for payload calibration in space applications.

## Figures and Tables

**Table 1:** LPFGs standard deviation values of repeatability measurements with optical interrogator.

Parameter	Standard DeviationLPFG1 (nm)	Standard Deviation LPFG2 (nm)	Standard Deviation Control LPFG (nm)
Temperature	0.02	0.03	0.01
Connectors	0.07	0.09	0.09
Weight	0.3	0.1	0.09
Setup	---	---	0.08

## Data Availability

Data is contained within the article.
